# SAMNA: accurate alignment of multiple biological networks based on simulated annealing

**DOI:** 10.1515/jib-2023-0006

**Published:** 2023-12-14

**Authors:** Jing Chen, Zixiang Wang, Jia Huang

**Affiliations:** School of Artificial Intelligence and Computer Science, Jiangnan University, Wuxi, China; Jiangsu Provincial Engineering Laboratory of Pattern Recognition and Computational Intelligence, Jiangnan University, Wuxi, China

**Keywords:** multiple network alignment, protein–protein interaction network, simulated annealing algorithm, network clustering, sequence similarity

## Abstract

Proteins are important parts of the biological structures and encode a lot of biological information. Protein–protein interaction network alignment is a model for analyzing proteins that helps discover conserved functions between organisms and predict unknown functions. In particular, multi-network alignment aims at finding the mapping relationship among multiple network nodes, so as to transfer the knowledge across species. However, with the increasing complexity of PPI networks, how to perform network alignment more accurately and efficiently is a new challenge. This paper proposes a new global network alignment algorithm called Simulated Annealing Multiple Network Alignment (SAMNA), using both network topology and sequence homology information. To generate the alignment, SAMNA first generates cross-network candidate clusters by a clustering algorithm on a *k*-partite similarity graph constructed with sequence similarity information, and then selects candidate cluster nodes as alignment results and optimizes them using an improved simulated annealing algorithm. Finally, the SAMNA algorithm was experimented on synthetic and real-world network datasets, and the results showed that SAMNA outperformed the state-of-the-art algorithm in biological performance.

## 1 Introduction

The rapid development of high-throughput screening technologies has resulted in an exponential increase in available molecular-level data, such as metabolic networks [1], gene regulatory networks [2], and protein–protein interaction (PPI) networks [3]. As the number of networks has gradually increased, so has the demand for analyzing and discovering hidden information in network data. Modeling known PPIs as network models and analyzing the spatial structure of protein complexes or proteins between different species are important references for inferring the functions of unknown proteins and predicting the evolutionary relationships of species [4, 5]. However, PPI networks are large in scale and complex in structure, making them more difficult to study by traditional methods, so that some scholars have proposed using network alignment algorithms to analyze them.

Network alignment constructs a mapping relationship among two or more nodes based on the network structure and node similarity. According to the number of networks to be aligned, alignment tasks can be divided into pairwise and multiple [6]. Pairwise network alignment is usually carried out between networks of species that are relatively well-studied and ones with incomplete studies [7]. Multiple network alignment is to match the networks of two or more species simultaneously, to find similar regions shared by multiple networks, and to obtain biological insights by making accurate network function predictions for species with missing or incomplete PPI networks [8]. Considering multiple related networks simultaneously can yield more useful information and thus facilitate node alignment, but the increased number of networks can make the computation rather complex [9]. In terms of the mapping of the results, network alignment tasks can also be divided into one to one, one to many, and many to many [10]. One-to-one alignment means that there is one and only one node from each network in the generated mapping relationship. One-to-many alignment means that a certain node in the source network can be mapped to multiple nodes in the target network, which is mostly used for metabolic path matching of the metabolic network [11]. If more than one node in one network mapping to multiple different nodes in another network, then the alignment is called as a many-to-many alignment. The global many-to-many network alignment strategy matches nodes with high conservation and similarity together. Aim is to extract protein clusters, where each cluster may include any number of proteins [12]. Multiple network alignment is to build clusters of mapping relationships among multiple networks, and each cluster may contain one or more nodes from a particular network, so multiple network alignment can produce either one-to-one or many-to-many alignment.

To date, various network alignment algorithms have been proposed. Many of these algorithms aim to find precise pairwise alignments between two networks, such as HubAlign [13], SANA [14], SAlign [15], CLMNA [16], and AligNet [17]. The study of multiple network alignments is growing and has resulted in a series of related algorithms, such as IsoRankN [18], multiMAGNA++ [19], BEAMS [20], NetCoffee [21], MPGM [22], and MONACO [23]. In particular, IsoRankN and multiMAGNA++ extend the pairwise network alignment algorithms IsoRank [24] and MAGNA++ [25], respectively, to multiple network alignment. BEAMS constructs *k*-partite graphs based on sequence similarity information and generates candidate clusters, and then uses greedy selection heuristics to obtain alignment with good biological consistency. NetCoffee is a global one-to-one multi-network alignment algorithm that calculates the topological similarity of nodes in different networks using the T-Coffee method, generates candidate matching node pairs by a maximal weight matching algorithm. Also, based on NetCoffee, the improved algorithms NetCoffee2 [26] and MAPPIN [27] have been proposed. The MPGM algorithm is a penetration-based graph matching algorithm that uses a seed-expansion strategy to generate many-to-many network alignment. MONACO is a recently proposed algorithm to find highly accurate alignment by iteratively and optimally matching local neighborhoods around focal nodes. The development of machine learning has also provided new ideas and methods that have benefited the task of network comparison. For example, Nasiri et al. [28] aim to present a modified version of Deepwalk based on feature selection, which benefits both network structure and protein features. ETNA [29] utilizes autoencoders to generate embeddings for each network, preserving the global and local topology of biological networks.

The goal of network alignment is to construct accurate and biologically meaningful results. Existing studies typically rely on the topological information of networks to maximize the similarity of comparison results. However, the complexity and diversity of PPI networks pose challenges for existing algorithms in discovering similar structures across different networks. Additionally, the complexity of biological processes contributes to issues such as missing data and data noise in species networks extracted through experimental methods, which significantly impact algorithm results. In this paper, we propose a new multi-network alignment algorithm called Simulated Annealing Multiple Network Alignment (SAMNA). SAMNA divides the network comparison process into two parts. The first part involves grouping nodes from different networks into distinct candidate clusters based on the similarity information of network sequences. This effectively addresses the problem of low similarity between candidate nodes and intermediate nodes encountered in existing algorithms. In the second part, SAMNA calculates a similarity score for different candidate clusters and utilizes an improved SA algorithm to iteratively solve the comparison results, aiming to retain as many similar nodes as possible. Experimental results using synthetic and real-world network datasets demonstrate that SAMNA outperforms existing algorithms in generating superior alignment results.

## 2 Methods

### 2.1 SAMNA algorithm

**Figure 1:**
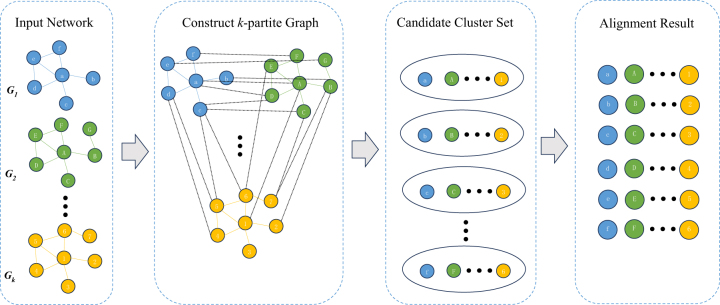
An illustrative example of SAMNA for node alignments. In the figure, *G*_1_, *G*_2_, …, *G*_k_ are the input networks, *V*_1_ = {a, b, c, d, e, f, g}, *V*_2_ = {A, B, C, D, E, F, G},…, *V*_*k*_ = {1, 2, 3, 4, 5, 6, 7} represent the nodes in their respective networks. In addition, bit scores of sequence similarity information serve as another input to the algorithm. Once the input is processed, the algorithm obtains the *k*-partite weighted undirected graph, candidate cluster set, and alignment result sequentially. The *k*-partite weighted undirected graph is constructed based on the sequence similarity information. A subgraph is generated for each node through the *k*-partite graph, and the *k*-clique with the highest weight is searched within the subgraph to form the candidate cluster with the node as the center. Subsequently, the candidate cluster set is obtained. Finally, the alignment results are selected from the candidate cluster set using the simulated annealing algorithm.

Firstly, SAMNA needs to input *k* networks $G_{1}=\left(V_{1},E_{1}\right),\ldots ,G_{k}=\left(V_{k},E_{k}\right)$ (*k* > 2) and sequence similarity information *Seq*, specifying *V*_*i*_ to denote the set of vertices (proteins) of the *i*th network and *E*_*i*_ to denote the set of edges (interactions) of the *i*th network, and construct a *k*-partite weighted undirected graph *M*_*α*_ based on the node sequence similarity information. For each node *u* in *V* = *V*_1_ ∪ *V*_2_ ∪ … ∪ *V*_*k*_, constructs a conservative subgraph *NG* with *u* as the core and consisting of its neighbor nodes. Subsequently, nodes with the maximum weight and in different networks in the subgraph *NG* are extracted as candidate clusters and added to the set of candidate clusters. Finally, search for alignment in the candidate cluster set and use the improved SA algorithm to optimize the alignment results. The specific process of the SAMNA algorithm is shown in Figure 1. Table 1 summarizes the notations frequently used in this paper.

**Table 1:** Summary of notations.

Notation	Definition
*G*	The unweighted and undirected graph
|*V*|	The number of vertices in *G*
|*E*|	The number of edges in *G*
*k*	Network numbers
*M*	A *k*-partite weighted graph
*A*	The final alignment
*C*_*i*_	The cluster of nodes containing nodes *i*

### 2.2 Delineating set of candidate clusters

In different species, conserved protein complexes should usually have homologous genes with high sequence similarity [30]. Therefore, we use sequence score *Seq* to construct a *k*-partite weighted graph *M*. The weight of an edge in the graph represents the sequence similarity score between the two nodes, which is obtained with the help of the Basic Local Alignment Search Tool (BLAST) [31]. Considering the large size of the *k*-partite graph *M*, it needs to be filtered, otherwise it will take a lot of time to directly compute the graph. *M*_*α*_ is a filtered version of the similarity graph *M*, which filters out some edges in *M* by a user-defined threshold *α*, such that the edge *E*_*u*,*v*_, where *u*, *v* ∈ *V*, for which $w\left(u,v\right)<\alpha \times \max\left(u,v\right)$ , will be deleted from *M*. Here, $w\left(u,v\right)$ denotes the edge weight in *M*, that is, the sequence similarity *Seq*_*u*,*v*_, and max(*u*, *v*) denotes the maximum value of the edge weight associated with *u* and *v* in *M*.

For node *u* in graph *M*_*α*_, where *u* ∈ *V*, a conservative subgraph *NG* consisting of node *u* and its neighbor nodes is constructed. This subgraph *NG* is then searched for the sub-cluster *C*_*u*_ that contains node *u* with maximum edge weight, where there is one and only one node from each network in *C*_*u*_. The maximum-weight clusters are generated and searched by a branch-and-bound algorithm, and the solution-space tree of the problem is searched by the breadth-first strategy [32].

### 2.3 Computing alignment scores

For matching multiple networks, our aim is to find an alignment result $\text{A}=\left\{C_{1},C_{2},\ldots ,C_{l}\right\}$ that maximizes the score of the defined objective function, where *C*_i_ represents the cluster $C_{i}=\left\{c_{1,i},c_{2,i},\ldots ,c_{k,i}\right\}$ , where *c*_*r*,*i*_ denotes the set of nodes from the *r*th network in the *i*th cluster in the alignment with *c*_*r*,*i*_ ∩ *c*_*r*,*j*_ = ∅, ∀*i* ≠ *j*, indicating that a node can appear in only one cluster.

To measure the quality of an alignment result, herein we use the CIQ and ICQ indicators proposed to formulate an objective function to measure the alignment. The function is defined as

(1) $$\begin{array}{c} S\left(A\right)=\alpha \text{CIQ}\left(A\right)+\left(1-\alpha \right)\text{ICQ}\left(A\right), \end{array}$$

where CIQ measures the topological quality between the alignment clusters, and ICQ measures the sequence score of node quality within a cluster and *α* ∈ [0, 1] is a balance parameter that determines the contribution of the network topology relative to the sequence similarity in the alignment process. The CIQ score of each alignment is defined as

(2) $$\begin{array}{c} \text{CIQ}\left(A\right)=\frac{\underset{\forall C_{m},C_{n}}{\sum }\left|E_{C_{m},C_{n}}\right|\times cs\left(m,n\right)}{\underset{\forall C_{m},C_{n}}{\sum }\left|E_{C_{m},C_{n}}\right|}, \end{array}$$

where *C*_*m*_ and *C*_*n*_ indicates the cluster of nodes containing nodes *m*, *n*, $E_{C_{m},C_{n}}$ is the set of edges connected to nodes in clusters *C*_*m*_ and *C*_*n*_, and $cs\left(m,n\right)$ is used to measure the conservative fraction of the cluster where node *m*, *n* is located, and the formula is as follows

(3) $$\begin{array}{c} cs\left(m,n\right)=\frac{{h^{\prime }}_{m,n}}{h_{m,n}}, \end{array}$$

where *h*_*m*,*n*_ is the number of PPI networks shared by the nodes in both *C*_*m*_ and *C*_*n*_, $h_{m,n}^{\prime }$ is the number of networks containing the edges in $E_{C_{m},C_{n}}$ . The ICQ score in Equation (1) is defined as

(4) $$\begin{array}{c} \text{ICQ}\left(A\right)=\frac{\underset{C_{i}\in A}{\sum }\text{ICQ}\left(C_{i}\right)}{\left|A\right|}, \end{array}$$

(5) $$\begin{array}{c} \text{ICQ}\left(C_{i}\right)=\frac{\underset{\forall \left(u,v\right)\in E\left(C_{i}\right)}{\sum }\sqrt{\frac{w{\left(u,v\right)}^{2}}{w_{\max}\left(u\right)\times w_{\max}\left(v\right)}}}{\left|\text{edge}\left(C_{i}\right)\right|}. \end{array}$$

Equation (4) calculates the overall ICQ score of all alignment clusters, and in Equation (5), *w*(*u*, *v*) is the sequence similarity score between nodes *u* and *v*, $w_{\max}\left(u\right)$ is the maximum value of the weights in the edges connected to node *u* in the similarity graph *M*_*α*_, and edge(*C*_*i*_) is the set of edges connected to the nodes in cluster *C*_*i*_ in the graph *M*_*α*_. In this way, ICQ computes the internal sequence similarity metric for a given cluster *C*_*i*_, thus representing the biological score of the whole alignment.

### 2.4 Simulated-annealing optimization alignment

Now, we perform an iterative optimization using the SA algorithm with an improved update, by using the generated candidate cluster set *CL* = ∑_*u*∈*V*_ *C*_*u*_ and the alignment scores. The SA algorithm must be initialized by setting an initial temperature parameter *T*_max_, the lowest temperature *T*_min_, the number of iterations *K*, and the cooling coefficient *s*.

Initially, the algorithm starts with an empty alignment, randomly selects a candidate cluster from the candidate cluster set *CL* generated in the previous step, and calculates the objective function score of the current alignment after joining that candidate cluster. If the objective function score of the new alignment increases compared to that of the previous iteration, then the new alignment is accepted unconditionally. However, if the difference between the objective function of the two alignment combinations decreases, then the probability of accepting the current alignment is calculated according to the metropolis criterion [26]. That is, an arbitrary value between zero and one is taken, and if this value is less than the probability of accepting the current solution, then the resulting new alignment is accepted; otherwise, the algorithm goes to the next loop and continues to loop until the temperature drops to a minimum value.

Because the candidate cluster of the SAMNA algorithm usually contains *n* (2 ≤ *n* ≤ *k*) nodes, the overlap of nodes during each state update is different. According to the number of overlapping nodes, it can be divided into fully overlapping, partially overlapping and no overlapping nodes. Herein, we improve the state update method of the NetCoffee2 algorithm [21]. The candidate cluster selected during each iteration is *C*′ = {*v*_1_, *v*_2_, …, *v*_*l*_}, where *l* ≤ *k*; the set of node clusters that have been matched is *A* = {*C*_1_, *C*_2_, …, *C*_*n*_}. When *C*′ ∩ *A* = ∅ is satisfied between the candidate clusters and the matched cluster set, the candidate cluster is added to the matched cluster set. When *C*′ ∩ *A* ≠ ∅, the update is divided into the following three situations and the updating algorithm is detailed in Algorithm 1 below.

(1) |*C*′ ∩ *A*| = |*C*′| indicates that all nodes in cluster *C*′ have overlap with current alignment *A*, which can be divided into two cases: the overlapping nodes are in the same cluster, or the overlapping nodes are scattered in multiple clusters. In both cases, the candidate clusters selected in the current iteration indicate that they have been matched, and these nodes are not considered.
(2) For a certain matched cluster *C*_*i*_ in *A* exists $\left|C^{\prime }\cap C_{i}\right|\geq \frac{1}{2}\left|C^{\prime }\right|$ ; When more than half of the nodes in the cluster overlap, it indicates that the correlation between two cluster is high, so the unduplicated nodes in cluster *c* are replaced with the nodes in cluster *C*_*i*_ and a new state solution are generated by adding local perturbations using the properties of simulated annealing. For a certain cluster *C*_*i*_ that has been matched in *A* there exists $\left|C^{\prime }\cap C_{i}\right|\geq \frac{1}{2}\left|C^{\prime }\right|$ ; That is, when more than half of the nodes in the cluster overlap with *C*_*i*_, which indicates a high correlation between the two clusters, so the unduplicated nodes in cluster *C*′ are replaced with the nodes in cluster *C*_*i*_ that are in the same network with them, and the new state solution is generated by adding local perturbations using the properties of simulated annealing.
(3) For the repeated nodes in cluster *C*′, if $\left|C^{\prime }\cap A\right|\leq \frac{1}{4}\left|C^{\prime }\right|$ , it means that the nodes in cluster are not highly correlated with the nodes in the matched cluster, and the overlap proportion of nodes is small, so the duplicate nodes in cluster *C*′ are removed, and the cluster formed by the remaining nodes is added to the candidate cluster for the next iterative search.

**Algorithm 1**

SAMNA

**Table:** 

**Input:** *Clusters CL*, *T*_max_, *T*_min_, *Iteration*, *s*
**Output:** A*: *best network alignment result*
1: *Let A* = {ø}, *i* = 0, *T*_0_ = *T*_max_
2: **while** *T*_*i*_ ≥ *T*_min_ **do**
3: *n* = 0
4: $T_{i}=T_{i}-\frac{i*\left(T_{\max}-T_{\min}\right)}{K}$
5: **while** *n* ≤ *Iteration* **do**
6: *randomly select C*_*i*_ ∈ *CL*
7: *A*′ = *update* (*A*, *C*_*i*_)
8: *compute* Δ *of S between A*′ *and A*
9: **if** Δ > 0 **then**
10: *A* = *A*′
11: **else**
*A* = *A*′ *if random*(0,1) < $\exp\left\{\frac{\Delta }{s*T_{i}}\right\}$
12: **end if**
13: *n* = *n* + 1
14: **end while**
15: *i* = *i* + 1
16: **end while**
17: A* = *A*
18: **return** A*;

## 3 Results and discussion

### 3.1 Datasets

To verify the effectiveness of the SAMNA algorithm proposed herein, it was tested on both synthetic and real-world networks. The synthetic networks used a database from Network Alignment Performance Assessment Benchmark (NAPAbench2) [33], which contains crystal growth (CG), duplication-mutation-complementation (DMC), and duplication with random mutation (DMR) synthetic-network data sets. Each model contained eight networks, and the numbers of nodes and edges of the PPI network were as given in Table 2.

**Table 2:** Numbers of proteins and interactions of synthetic networks.

Dataset	CG		DMC		DMR	
	Node	Edge	Node	Edge	Node	Edge
A	1000	3985	1000	1919	1000	2031
B	1000	3985	1000	1853	1000	2092
C	1000	3985	1000	1923	1000	1967
D	1000	3985	1000	1840	1000	1977
E	1000	3985	1000	1867	1000	1959
F	1000	3985	1000	1848	1000	1998
G	1000	3985	1000	1818	1000	2030
H	1000	3985	1000	1867	1000	2056

The real-world networks used five eukaryotic networks derived from the IsoBase [34] database, i.e., *Saccharomyces cerevisiae*, *Caenorhabditis elegans*, *Drosophila melanogaster*, *Homo sapiens*, and *Mus musculus*, and the numbers of nodes and edges of the PPI network were as given in Table 3. The sequence similarity information of nodes between different species came from the bit score generated by BLAST obtained on Ensembl [31].

**Table 3:** Numbers of proteins and interactions of five eukaryotic species.

	Node	Edge
*Saccharomyces cerevisiae*	5524	164,718
*Caenorhabditis elegans*	2995	8639
*Drosophila melanogaster*	7396	49,467
*Homo sapiens*	10,403	105,232
*Mus musculus*	623	776

### 3.2 Experimental parameters

To validate the experimental results, we selected and compared three widely used multi-network alignment algorithms that also provide source code: IsoRankN [18], NetCoffee [21], NetCoffee2 [26], and ACCMNA [35].

During the experiment, the parameters of the selected algorithms were set to the values recommended in their respective papers. Additionally, SAMNA only has one adjustable parameter, *α*. In the synthetic-network experiment, we used *α* = 0.2, while in the real-network experiment, due to the relatively high network noise, we used *α* = 0.8. The hardware parameters of the actual operating environment used in this study are presented in Table 4.

**Table 4:** Algorithm operating environment.

Operating System	CPU	Memory
Linux	Intel^®^ Core ™ i5-7500 CPU @ 3.40 GHz	32 G
Windows	Intel^®^ Core ™ i7-12700 CPU @ 2.10 GHz–4.9 GHz	16 G

### 3.3 Evaluation of adjustable parameters

**Figure 2:**
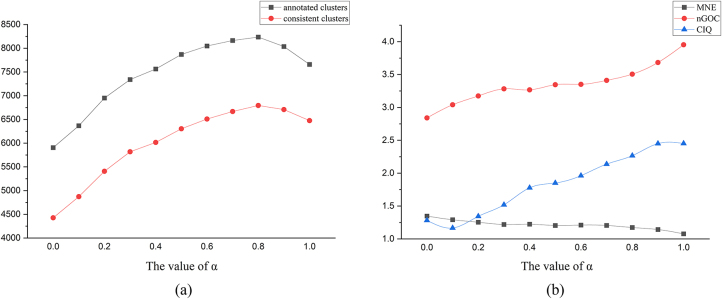
Evaluation of alignment results under different settings of *α* parameter.

As shown in Figure 2(a), the values of annotated clustering and consistent clustering increase initially with increasing *α* and then start to decrease when *α* reaches 0.8. In Figure 2(b), the results for nGOC and CIQ show an overall zigzag upward trend with increasing *α*, and the value of MNE decreases; this indicates that nGOC, CIQ, and MNE all improve with increasing *α*. Also, as is predictable, the running time of the algorithm decreases with increasing *α*. Taken together, the value of *α* for the experiment on the real-world networks was taken as 0.8.

We evaluate the quality of the alignment results obtained by different multi-network algorithms from the following indicators: annotated clusters, consistent clusters, mean normalized entropy (MNE) [18], specificity (SPE), normalized gene ontology (GO) consistency (nGOC), and cluster interaction quality (CIQ) [20]. A consistent cluster means that all proteins in the cluster are annotated by at least one general standard GO. SPE is the ratio of consistent clusters to annotated ones, where an annotated cluster means that at least two nodes in the cluster are annotated by GO. MNE and nGOC are the consistency evaluation indicators proposed in IsoRankN [18] and BEAMS [20], respectively, which offer a good measure of the biological quality of the alignment results; the lower the value of MNE, the more coherent is a cluster. Each of the four aforementioned indicators is a biological indicator, and the topological quality of the alignment results can be measured by CIQ, which indicates how conservative the edges between two clusters are in a given alignment.

SAMNA uses the parameter *α*, which is a threshold parameter with values between zero and one. Here, we assess the influence of *α* on our experimental results on the real-world network data.

### 3.4 Alignment results on synthetic networks

**Figure 3:**
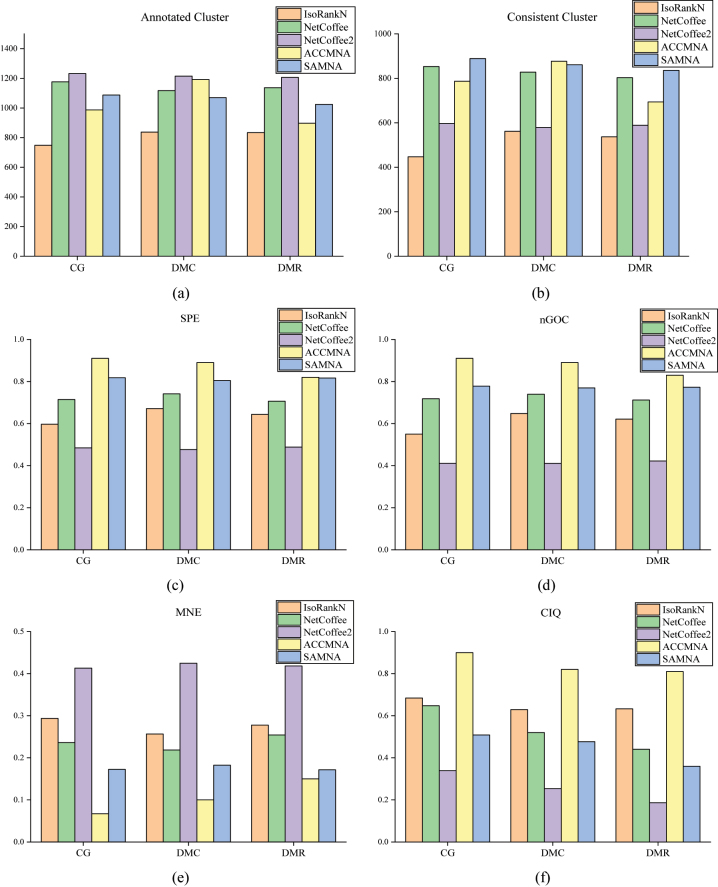
Experimental performance on synthetic network. (a) Annotated cluster and (b) consistent cluster of the different algorithms on synthetic networks, where the horizontal coordinate is the dataset and the vertical coordinate is the number of indicators. (c) SPE, (d) nGOC, (e) MNE and (f) CIQ of the different algorithms on synthetic networks, where the horizontal coordinate is the dataset and the vertical coordinate is the score of indicators.

The experimental results on the synthetic networks are shown in Figure 3. Figure 3(a) displays the annotated-cluster coverage of the alignment results obtained from NetCoffee, NetCoffee2, IsoRankN, ACCMNA, and SAMNA on the CG, DMC, and DMR synthetic-network datasets. It is evident that NetCoffee2 generated the highest annotated-cluster coverage among the five algorithms. Figure 3(b) illustrates the consistent cluster coverage produced by the five algorithms on the synthetic network dataset. The results indicate that SAMNA exhibits the highest consistent cluster coverage among the algorithms on CG and DMR, and it is only slightly lower than ACCMNA on DMC. Figure 3(c) and (d) present the SPE and nGOC results for the five algorithms. It is observed that SAMNA achieved the second highest scores across the three synthetic network datasets. Figure 3(e) shows the MNE results. It is seen that the MNE values of SAMNA are much lower than those of IsoRankN, Netcoffee and NetCoffee2, which indicates that the alignment results achieved a better biological consistency. Figure 3(f) shows the experimental results of topological index CIQ; we can see that the topology of ACCMNA is the best, NetCoffee2 performs the worst and SAMNA performs average.

Experiments of the SAMNA algorithm on synthetic networks verified its theoretical feasibility. Experimental results on the CG, DMC, and DMR synthetic network datasets show that SAMNA produces the highest consistent cluster coverage among the five algorithms, i.e., it produces more biologically meaningful clusters. Meanwhile, SAMNA demonstrated better biofunctional SPE alignment scores on the three synthetic network datasets, indicating a higher proportion of equivalent clusters compared to the other four algorithms. SAMNA also performed well on the biological consistency indices MNE and nGOC. The ACCMNA algorithm also obtained higher scores on these indices, but its use of a greedy algorithm for generating comparison clusters in each iteration makes it susceptible to local optimality issues. In terms of the scores on the topological index CIQ, both ACCMNA and IsoRankN yielded better results across the three datasets. This is because IsoRankN and ACCMNA produce many-to-many aligned results, so the conservative edge rate between clusters is high. However, compared to the other three algorithms, IsoRankN has poor results in terms of biological features, which is because IsoRankN focuses too much on the topology of the network and ignores the biological features in generating the alignments. This gives it good topological quality, but poor results for biologically meaningful alignments.

Collectively, the experiment with the SAMNA algorithm on synthetic networks verified its theoretical feasibility. The results on the three synthetic-network datasets show that SAMNA performs well experimentally on synthetic networks and outperforms other comparative algorithms in some results.

### 3.5 Alignment results on real-world network

The synthetic-network structure was relatively ideal and was used only for validating the SAMNA algorithm theoretically. To further verify the reliability of the algorithm, we also verified it on real-world networks; this offers a more complex situation because the size of the real-world networks varied greatly and the protein data were incomplete.

**Figure 4:**
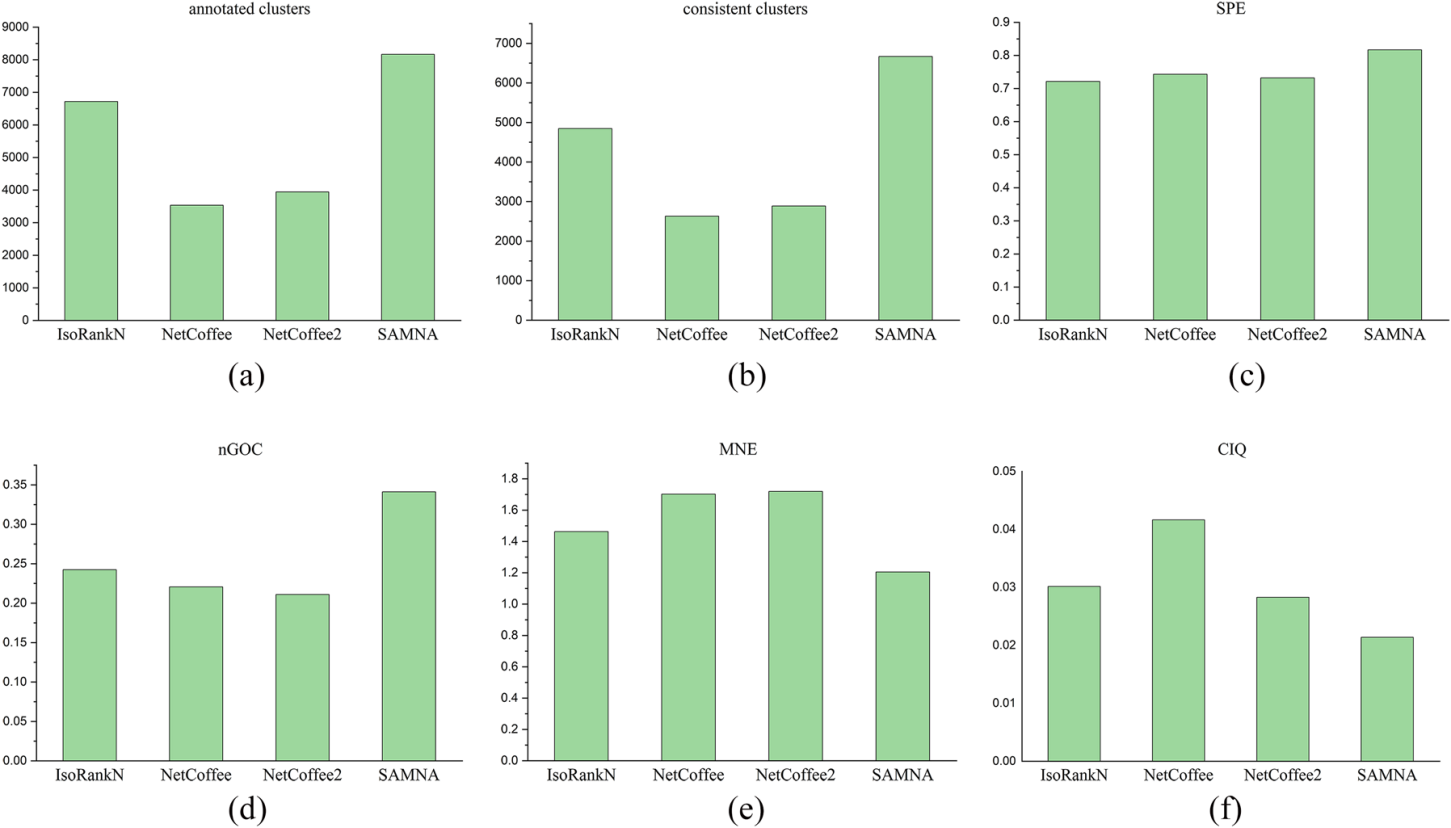
Experimental performance on real-world network. (a) Annotated cluster and (b) consistent cluster of the different algorithms on real-world networks, where the horizontal coordinate is the algorithms and the vertical coordinate is the number of indicators. (c) SPE, (d) nGOC, (e) MNE and (f) CIQ of the different algorithms on real-world networks, where the horizontal coordinate is the algorithms and the vertical coordinate is the score of indicators.

Figure 4 shows the experimental results of the different algorithms on the real-world networks. Figure 4(a) and (b) show the annotated-cluster and consistent-cluster coverages, respectively. As can be seen, of the four algorithms, SAMNA produced the best annotated and consistent clustering on the real-world networks, indicating that it produces better alignment under a complex network structure. Figure 4(c)–(f) present the experimental results of the topological and biological evaluation metrics for the five algorithms on real-world networks. In terms of the CIQ scores for topological metrics, NetCoffee achieves the highest score, while SAMNA obtains the lowest score. However, there is no significant difference in the scores between SAMNA, IsoRankN, and NetCoffee2. On the other hand, SAMNA demonstrates the best results for the biofunction-related metrics, namely SPE, nGOC, and MNE. These results are significantly better compared to IsoRankN, NetCoffee, NetCoffee2, and ACCMNA algorithms.

Real-world networks are more complex and require algorithms with higher stability. It is noticeable that the ACCMNA algorithm, which performs well on synthetic networks, does not yield optimal results on real-world networks. This is because ACCMNA employs a greedy strategy that is highly influenced by noise present in the PPI network, leading to a local optimum. In contrast, SAMNA plays a more stable role, outperforming ACCMNA in this context. Overall, the alignment results generated by SAMNA are biologically more meaningful than those generated by the other algorithms, both on the synthetic-network datasets and on the more complex real-world network datasets.

## 4 Conclusions

Herein, we proposed SAMNA, a global one-to-one multiple-network alignment algorithm based on SA, which aims to generate biologically meaningful alignments through the topology of networks with sequence information. Methodologically, SAMNA uses a clustering algorithm to generate candidate cluster sets in a *k*-partite graph constructed from sequence similarities, and then it searches the candidate sets and optimizes the search results using SA algorithms.

Experimental results on synthetic and real-world networks verified the feasibility and effectiveness of SAMNA. Also, comparing it with other algorithms, SAMNA produced better results for alignment with biological significance. However, SAMNA lacks consideration of the network topology in the method of generating initial candidate clusters and does not exploit the similarity of nodes among network structures, which will be a focus of future work. Moreover, SAMNA algorithm only draws on Blast scores in using biological information, proteins have many other rich information, such as secondary structure motifs, 3D structural similarity, semantic similarity (using gene ontology) and phylogenetic information, etc., which will also be tried to use applied information to predict cluster similarity scores and improve the performance of the algorithm at a later stage.

## 5 Discussion

SAMNA produced better biofunctional SPE alignment scores on the three synthetic network datasets, indicating that it produced a high proportion of equivalent clusters among the four algorithms; SAMNA also scored well on the biological consistency indices MNE and nGOC. the ACCMNA algorithm also scored better on these indices, but the algorithm uses a greedy algorithm to generate comparison clusters in each iteration, which is prone to the problem of local optimality. In terms of the scores on the topological index CIQ, ACCMNA and IsoRankN produce better results on the three datasets.

## Supplementary Material

SAMNA is available at https://github.com/Toxiang/SAMNA.
